# B-MYB Is Essential for Normal Cell Cycle Progression and Chromosomal Stability of Embryonic Stem Cells

**DOI:** 10.1371/journal.pone.0002478

**Published:** 2008-06-25

**Authors:** Kirill V. Tarasov, Yelena S. Tarasova, Wai Leong Tam, Daniel R. Riordon, Steven T. Elliott, Gabriela Kania, Jinliang Li, Satoshi Yamanaka, David G. Crider, Gianluca Testa, Ronald A. Li, Bing Lim, Colin L. Stewart, Yie Liu, Jennifer E. Van Eyk, Robert P. Wersto, Anna M. Wobus, Kenneth R. Boheler

**Affiliations:** 1 Laboratory of Cardiovascular Science, National Institute on Aging, National Institutes of Health, Baltimore, Maryland, United States of America; 2 Stem Cell and Developmental Biology, Genome Institute of Singapore, Singapore, Singapore; 3 Department of Medicine, Johns Hopkins University, Baltimore, Maryland, United States of America; 4 In vitro Differentiation Group, Leibniz Institute of Plant Genetics and Crop Plant Research, Gatersleben, Germany; 5 Laboratory of Stem Cell Engineering & Bioelectricity, Stem Cell Institute, University of California Davis, Davis, California, United States of America; 6 Harvard Institutes of Medicine, Harvard Medical School, Boston, Massachusetts, United States of America; 7 Cancer and Developmental Biology Laboratory, National Cancer Institute, Frederick, Maryland, United States of America; 8 Laboratory of Molecular Genetics, National Institute on Aging, National Institutes of Health, Baltimore, Maryland, United States of America; 9 Flow Cytometry Group, National Institute on Aging, National Institutes of Health, Baltimore, Maryland, United States of America; Baylor College of Medicine, United States of America

## Abstract

**Background:**

The transcription factor B-Myb is present in all proliferating cells, and in mice engineered to remove this gene, embryos die in utero just after implantation due to inner cell mass defects. This lethal phenotype has generally been attributed to a proliferation defect in the cell cycle phase of G1.

**Methodology/Principal Findings:**

In the present study, we show that the major cell cycle defect in murine embryonic stem (mES) cells occurs in G2/M. Specifically, knockdown of B-Myb by short-hairpin RNAs results in delayed transit through G2/M, severe mitotic spindle and centrosome defects, and in polyploidy. Moreover, many euploid mES cells that are transiently deficient in B-Myb become aneuploid and can no longer be considered viable. Knockdown of B-Myb in mES cells also decreases Oct4 RNA and protein abundance, while over-expression of B-MYB modestly up-regulates *pou5f1* gene expression. The coordinated changes in B-Myb and Oct4 expression are due, at least partly, to the ability of B-Myb to directly modulate *pou5f1* gene promoter activity in vitro. Ultimately, the loss of B-Myb and associated loss of Oct4 lead to an increase in early markers of differentiation prior to the activation of caspase-mediated programmed cell death.

**Conclusions/Significance:**

Appropriate B-Myb expression is critical to the maintenance of chromosomally stable and pluripotent ES cells, but its absence promotes chromosomal instability that results in either aneuploidy or differentiation-associated cell death.

## Introduction

Avian myeloblastosis viral oncogene homolog-2 (*mybl2*) is a member of a multigene family of transcription factors involved in control of cell cycle progression, differentiation and apoptosis [1], [2]. All members of this family, A-MYB, B-MYB (MYBL2) and C-MYB, contain conserved regulatory and transactivation domains that exhibit sequence-specific DNA-binding activity [3]; and in vitro, each protein can bind to the same consensus sequence C/TAACNG [1]. B-MYB is, however, a relatively poor transactivator when compared with either A- or C-Myb. Among tissues, C-MYB is most prevalent in bone marrow and is critical to generation of definitive hematopoietic stem cells [4]; whereas, A-MYB is abundant in testes and is implicated in normal spermatogenesis and in mammary gland proliferation [5]. Only B-MYB, the ancestral gene of this family, is expressed in all proliferating cells [6].

As with many cell cycle associated transcription factors, B-Myb expression and function is dynamically regulated. The *mybl2* gene, which encodes B-Myb, is regulated directly by E2F transcription factors and is maximally induced at the G1/S boundary of the cell cycle [2], [7]. The *trans*-activation and gene regulatory potential of B-MYB is regulated by cyclin A/cdk2-mediated phosphorylation [8], and B-MYB is degraded through a ubiquitin-mediated process late in S phase [9]. Because it is prevalent during both late G1 and early S phases, the primary functions of B-MYB have generally been assumed to be restricted to these portions of the cell cycle [2], [7], [10]; however, studies in Drosophila [11], [12] and zebrafish [13] have shown that B-Myb is also implicated in chromosomal condensation, chromosomal stability and cell cycle progression through G2/M.

Importantly, B-Myb is the only member of this multigene family known to be present in embryonic stem (ES) cells, and developmentally, knockout of this gene results in embryonic lethality by 6.5 dpc in mice. Essentially this transcription factor is thought to be necessary for continued growth of the inner cell mass during the post-implantation phase of development [14]. Although the mechanism responsible for this lethality has generally been attributed to a defect in cell cycle progression through G1, based on studies using a dominant-negative and inducible form of C-Myb [15], neither the molecular basis of embryonic lethality nor its function in ES cells has been evaluated. In the present study, we show that B-MYB is required not only for normal ES cell cycle progression but also for correct mitotic spindle formation and maintenance of euploidy. Moreover, knockdown of B-Myb in ES cells promotes either differentiation coupled to apoptosis or generation of aneuploid ES cells.

## Results

### *Mybl2* gene products in pre-implantation blastocysts and pluripotent stem cells

**Figure 1:**
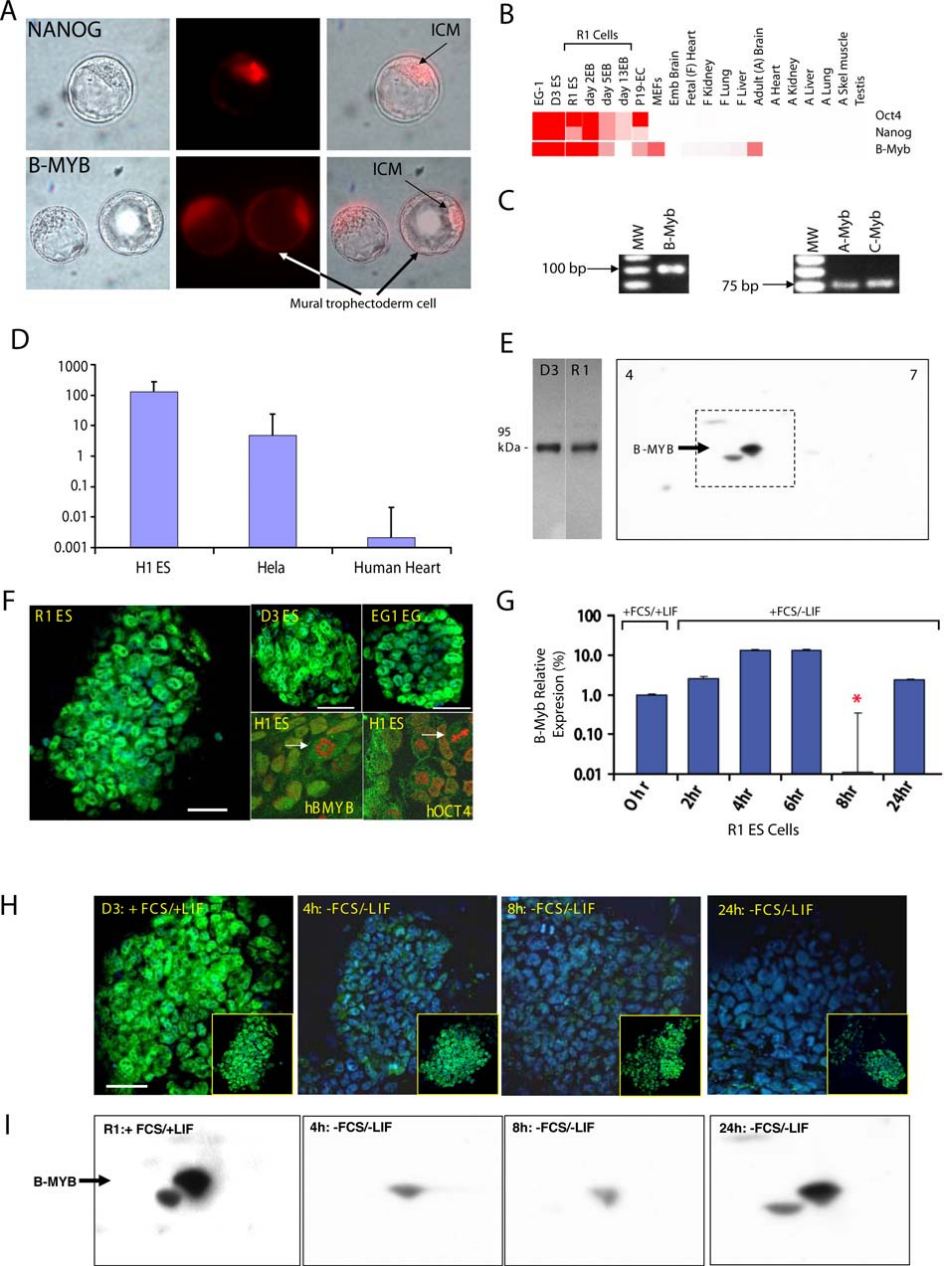
*Mybl2* gene products are abundant in pluripotent stem cells and are dynamically regulated during differentiation. A) Representative immunostainings of pre-implantation mouse blastocysts. Immunostaining of B-MYB proteins was observed not only in the inner cell mass (ICM) but also in the mural trophectoderm. Antibodies to NANOG were used to specifically mark cells of the ICM [42]. B) Summary qRT-PCR data showing the relative abundance of B-Myb mRNA among cells and tissues. Data are expressed in heat map form, where color intensity (red) indicates the relative abundance of B-Myb transcripts. Although relatively high expression is seen in proliferating MEFs and adult brain, transcripts are more than 100-fold less than that seen in ES cells. Data for Oct4 and Nanog are included for comparison. C) Transcripts for B-Myb (100 bp), A-Myb (74 bp) and C-Myb (76 bp) were detectable in ES cell lines by PCR, but B-Myb was the most abundant. D) Graphic representation of qRT-PCR analysis of B-Myb transcripts in hES cells (H1), HeLa cells and adult human tissues (heart). Similar to mES cells, B-Myb is highly abundant in hES cells. E) Representative one- and two-dimensional B-Myb immunoblot showing that anti-B-Myb antibodies react with a predicted protein of ∼95 kDa. F) B-MYB immunostaining is located in the nucleus, but during mitosis, it segregates away from chromatin, suggesting that its binding is dynamic. Data are from mouse lines (R1, D3 and EG-1), where B-Myb immunstaining is shown green (FITC) and nuclear staining (Hoechst) is in blue; and from human H1 ES cells, where immunostaining for B-Myb and Oct4 are shown in green (FITC), and nuclear staining is in red (TOPRO). A magnified view of human ES cells showing the dissociation of B-Myb and Oct4 from the chromatin of mitotic cells is indicated by arrows. G) B-Myb transcripts are transiently down-regulated when ES cells are induced to differentiate by serum (FCS) and LIF withdrawal. H) B-MYB immunostaining is transiently decreased by FCS and LIF withdrwal in D3 and R1 (inset) ES cells. I) The transient decrease in B-MYB proteins observed through immunostaining was quantitatively confirmed by western blotting. In this image, an expanded view of the two-dimensional westerns shown in Figure 1E (dashed boxed region) is shown. When normalized to total protein, the transient loss of B-MYB (two-spots) was highly reproducible (p<0.05) and similar to but more rapid than that observed for B-Myb RNA. Bar = 50 µm. *p<0.05 relative undifferentiated ES cells.

Previous studies have shown that B-Myb is present in mES cells, is required for continued epiblast growth during the post-implantation phase of mouse development, and is necessary for the derivation of mES cell lines [14]. We have extended these earlier studies and found that B-MYB proteins are present in pre-implantation embryos at 2- and 4- cell stages, in the morula and in early cavitation stage embryos (not shown). We also detect B-MYB in both the inner cell mass and mural trophectoderm of pre-implantation blastocysts (Figure 1A). Although it is unclear if B-MYB is functional at these embryonic stages, its early expression and presence in the developing trophectoderm suggest that it might have a regulatory role prior to implantation.

By qRT-PCR, we determined that B-Myb transcripts are from 100- to >10,000-fold more abundant in mES, murine embryonic germ (mEG), and murine embryonic carcinoma (mEC) cell lines than in all other fetal and adult mouse cell lines and tissues examined (Figure 1B). Transcripts to A-Myb and C-Myb were detected in mES and mEG cells (Figure 1C), but at levels much lower than those found in testes and Sca1^+^ bone marrow cells, respectively. In contrast, B-Myb was ∼10- and 5-fold less abundant in testes and Sca1^+^ cells than were transcripts to A-Myb and C-Myb, respectively (n≥3 independent samples). The relative abundance of *myb* family members in these tissues is consistent with the expression data found in the Gene Expression Atlas, thus confirming primer specificity and the presence of A- and C-Myb transcripts in ES cells. Interestingly, the quantity of A-Myb and B-Myb transcripts were similar in trophectoderm (TS) cell lines. Finally, B-Myb transcripts were also prevalent in the human ES cell line H1 and at levels much greater than in normal human adult tissues like heart. Its abundance was, however, only 5–10-fold greater on average than that in HeLa cells, which has relatively high levels of B-Myb (see http://symatlas.gnf.org/SymAtlas/201710_at in NCI60 on U133A, gcRMA)(Figure 1D).

In mES cells, a band (spot) of 95 kDa was observed for B-MYB on both one- and two-dimensional gel western blots (Figure 1E). No signal for either A- or C-MYB could however be demonstrated in any ES cell line (R1, D3) examined, thus confirming the preponderance of B-MYB in mES cells. At the cellular level, B-MYB immunostaining was predominantly nuclear and relatively homogeneous in mES and in human ES cells (line H1) (Figure 1F). Mitotic cells, however, had a unique staining pattern that was characterized by dissociation of B-MYB and OCT4 from the chromatin (Figure 1F, hBMYB and hOCT4). This latter finding indicates that these transcription factors may be redistributed to the extrachromosomal space during periods of mitosis.

Notably, induction of mES cell differentiation led to dynamic changes in B-Myb RNA and protein expression, but the timing of change depended on the model of differentiation. The first model that we tested consisted of an aggregation technique where mES cells were dissociated with trypsin and allowed to reaggregate to form embryoid bodies (EB) in the absence of leukemia inhibitory factor (LIF). With this model, B-Myb RNAs were significantly reduced within 48–72 hours (See Figure 1B, R1 Cells), and this reduction was slightly earlier than the decrease in Oct4 transcripts. Separately, withdrawal of both fetal calf serum (FCS) and LIF from non-aggregated mES cells led to a significant decrease in B-Myb transcripts within 8 hours of withdrawal. This decrease was transient, because within 24 hours, B-Myb transcripts returned to control levels (Figure 1G). No significant change in Oct4 transcripts could however be demonstrated during this same time course. At the cellular level, FCS and LIF withdrawal induced a rapid decrease in B-MYB immunostaining in mouse ES cells (D3, R1: Figure 1H). The reduction was particularly apparent in D3 ES cells, where loss of staining was observed within 2 hours and prior to any significant change in B-Myb RNA. The greatest reduction in immunostaining was observed at 8 hours of differentiation, and similar to the RNA data, the signal intensity returned to baseline levels at ≥24 hours after FCS and LIF withdrawal. Moreover, these dynamic and transient changes in B-MYB protein abundance could be quantified by western. B-MYB proteins significantly (p<0.05) decreased (relative to total protein) within 4 to 8 hours of FCS and LIF withdrawal, but after 24 hours, the protein returned to levels observed in undifferentiated cells (Figure 1I).

### Knock-down of B-Myb

**Figure 2:**
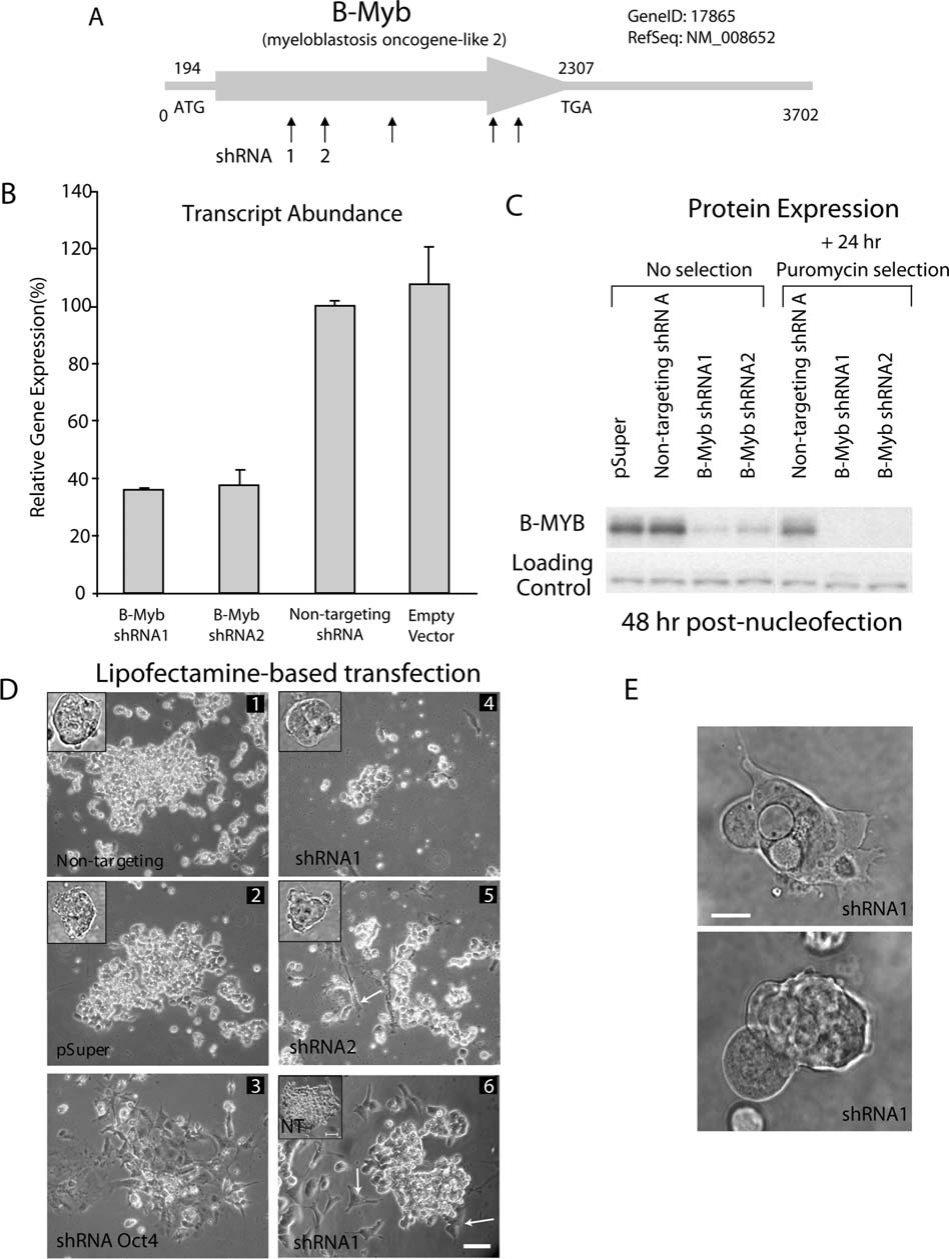
shRNAs specifically knockdown B-Myb in ES cells. A) Line drawing of B-Myb and location of silencing shRNAs tested in this study. B) Endogenous B-Myb mRNAs are effectively reduced following cell transfections with shRNAs against B-Myb (shRNA1, shRNA2), but no reduction in B-Myb could be demonstrated with the negative controls [non-targeting (NT) shRNAs or empty vector (pSuper.puro)]. C) By western, B-MYB proteins were reduced in ES cells by ∼50–70% following knockdown with shRNA1 or shRNA2, but when selected with puromycin, reductions in B-MYB of >90% were routinely seen. Loading controls indicate that the shRNAs were specific for B-Myb. D) Representative cell morphologies of R1 and D3 mES cells after transfection. The insets in panels 1, 2, 4 and 5 correspond to cell morphologies of R1 cells 24 hours after nucleofection and prior to puromycin selection. D3 cells are also shown 48 hours (Panels 1–5) and 72 hours (Panel 6) after lipofection and puromycin selection. The shRNAs employed in each set of experiments are indicated in the figure, and consisted of negative controls (Non-targeting, NT or pSuper), a positive control (shRNA specific for Oct4) and experimentals (shRNA1 and 2, which are specific for B-Myb). Knockdown of B-Myb effectively reduced colony sizes (Panels 4, 5) and led to cells with a more differentiated phenotype (Panels 5 and 6, see arrows). In panel 6, a non-targeting (NT) control is included for comparisons. Size bar = 50 µm. E) Phase image of R1 ES cells at 72-hours post nucleofection. Cells lacking B-Myb (shRNA1) and selected by puromycin for 48 hours showed distinct signs of differentiation, vacuolization and membrane blebbing. Bar = 25 µm.

Since B-Myb was transiently down-regulated in mES cells with differentiation, a short-hairpin RNA based technique was employed to deplete B-Myb mRNAs to examine its function. Five short-hairpin RNAs (shRNAs) were cloned into pSuper.puro (Figure 2A, and Table 1) and transfected into E14 and D3 or nucleofected into R1 and D3 ES cell lines. In pilot experiments, all five shRNAs decreased the quantity of B-Myb in the E14 and D3 cells, but shRNA1 and 2 proved most effective. In this study, only these latter two constructs were employed to knockdown B-Myb. A non-targeting (NT) shRNA and empty vector (pSuper) were employed as negative controls, and a shRNA to Oct4 was used as a positive control.

**Table 1:** shRNA sequences

shRNA Construct	Target Sequence	Position
B-Myb RNA1	GGTGCGACCTGAGTAAATT	1159–1177
B-Myb RNA2	GAAGAGCACGCCTGTCAAA	1526–1544
B-Myb RNA3	GTCTCTGGCTCTCGACATT	1958–1976
B-Myb RNA4	GTAACAGCCTGCTCAACCA	2086–2104
B-Myb RNA5	GGTGTGGGGTAAGGTTAAA	2354–2372
NT RNA	GTAAGGGAGATGAGGCAAA	

Gene-specific regions for RNA interference were designed as described in the text and double stranded oligonucleotide cassettes were cloned into pSUPER.puro (Oligoengine) after BLAST analysis to ensure against sequence similarity with other genes. Positions are based on |NM008652.2| *Mus musculus* myeloblastosis oncogene-like 2 (Mybl2), mRNA. NT- non-targeting.

Here, we present data from both lipofectamine-transfected (D3) and nucleofected (R1) ES cells; however, identical expression data were observed with transfected E14 and nucleofected R1 and D3 ES cells, which rules out any possibility of major variations in cellular responses due to cell line variation or to the mode of transfection. Following transfection, cells were cultivated in the presence of FCS and LIF, prior to the selective addition of puromycin to enrich for transfected cells. Relative to the NT shRNA controls, cells transfected with control Oct4 shRNA reduced endogenous Oct4 transcripts by >90% (data not shown), while knock-down of B-Myb led to a significant reduction in endogenous B-Myb transcripts by 60–70% (Figure 2B) and by >90% in puromycin-selected cells (Figure 2C). None of the major proteins observed on an amido black stained gel showed altered expression and, more specifically, no change in Utf1 and Hdac transcripts could be seen (see below), demonstrating the specificity of this shRNA-mediated knockdown.

After an overnight cultivation, nucleofected R1 mES cells formed small dome-shaped colonies that consisted primarily of small round cells with a relatively large nuclear to cytoplasmic ratio (Figure 2D, insets in panels 1, 2, 4 and 5). The lipofectamine treated cells however formed loosely aggregated, flattened cell clusters. We attribute the morphological changes to differences between the two protocols, but importantly and irrespective of the technique, puromycin-resistant ES cells colonies transfected or nucleofected with control vectors (pSuper, NT) remained relatively compact and increased in size as a function of time (Figure 2D, panels 1 and 2). Selected cells that had been transfected with a shRNA against Oct4 however began to differentiate. Specifically, most of the Oct4-deficient cells appeared relatively large and had distinct cytoplasmic and nuclear regions (Figure 2D, panel 3). Similarly, knockdown of B-Myb led to a modified colony morphology, but one that was distinct from cells lacking Oct4 (Figure 2D, panels 4–6). First, the puromycin-resistant ES cells lacking B-Myb were comprised primarily of small, and apparently non-proliferating cell clusters. Second and within 48–72 hours of transfection, a number of cells both within and around the cell clusters began to exhibit characteristics of differentiated cells (Figure 2D, panels 5 and 6, see arrows). These included the formation of spindle shaped cells, and an increase in the cytoplasmic to nuclear ratio. Most of the cells within a cluster/colony, however, retained morphological traits consistent with undifferentiated ES cells. Subsequently and at >72 hours post-puromycin selection, numerous individual and small groups of cells were present that showed distinct signs of differentiation (finger-projections, low nuclear to cytoplasm ratio), vacuolization and membrane blebbing (Figure 2E). Altogether, these data suggest that B-Myb might play a role not only in mES cell proliferation but also in the initiation of differentiation.

### Loss of B-Myb inhibits proliferation, increases aneuploidy and promotes mitotic spindle errors

**Figure 3:**
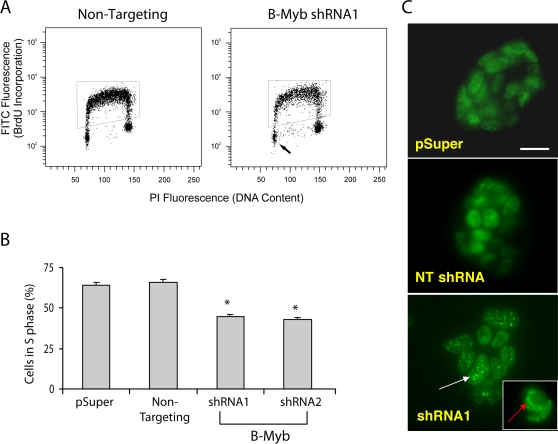
BrdU incorporation into R1 ES cells. A) Representative flow cytometry data of nucleofected cells that were cultivated for 24 hours, pulse labelled with BrdU for 30 minutes and immunostained with an antibody specific for BrdU. FITC fluorescence of BrdU stained cells is shown in the boxed region (i.e., S phase cells), and the arrow points to the region of the histogram corresponding to cells in G0/G1. B) Averaged data showing the number of cells in S phase decreased following knockdown of B-Myb (n = 3, *p<0.05 relative to NT controls). C) Down-regulation of B-Myb leads to a defect in BrdU incorporation during S phase. Immunostained control cells (NT shRNA or pSuper) that incorporated BrdU for 2 hours had a relatively homogeneous but cloudy pattern of staining; whereas, cells lacking B-Myb (shRNA1) displayed a unique pattern characterized by punctate and/or patchy staining (white arrow, p<0.001). The inset (red arrow) shows one group of cells that appears to have inappropriate chromosomal structure. Bar = 20 µm.

To directly assess proliferation, shRNA nucleofected cells were exposed to bromodeoxyuridine (BrdU) and incorporation of this thymidine analog into unsynchronized R1 ES cells was quantified by flow cytometry (Figure 3A). In these experiments, non-selected ES cells were examined 48 hours after nucleofection, and following pulse incorporation of BrdU (30-minutes) into newly synthesized DNA to provide an estimate of the fraction of cells in S-phase. The percentages of BrdU-labeled cells decreased in cells transfected with shRNAs targeted to B-Myb relative to controls (Figure 3B, p<0.001). At this time, the number of cells in G1, determined by propidium iodine incorporation, did not differ among any of the nucleofected groups, but a significant increase and decrease, respectively in the number of cells in G2/M and S phases were observed in B-Myb-deficient cells. Additionally, a two-hour pulse produced a highly significant (p<0.001) and unique pattern of BrdU incorporation, characterized by localized, patchy or punctate staining. The percentage of cells (n≥200) with strong punctate or patchy staining ranged from 35% (pSuper, NT) to 67% (shRNA1); whereas, those cells with a more diffuse or relatively homogenously BrdU staining (containing discrete foci of BrdU immunoreactivity equally distributed throughout the nucleus) ranged from 65% in controls to 32% in knockdown experiments (Figure 3C). This pattern of incorporation is consistent with either changes in chromosome condensation, similar to those observed in developing Drosophila eye [12] or, alternatively, to altered cell cycle states in S phase.

**Figure 4:**
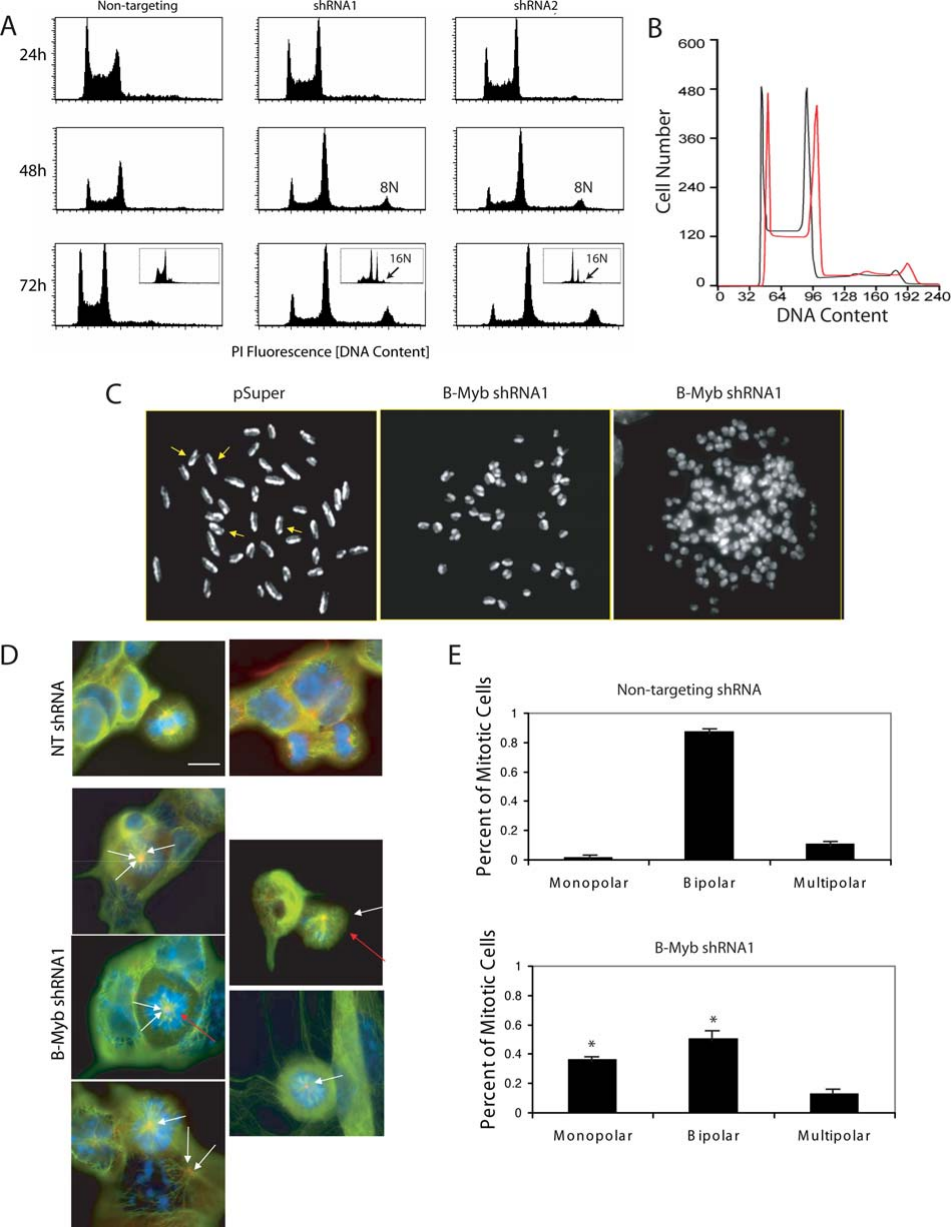
DNA analysis of R1 ES cells. A) DNA content was measured by flow cytometry in non-synchronized ES cells that had been fixed and stained with propidium iodine following nucleofection with shRNA. At 24 hours after nucleofection with shRNA1 and 2, the number of cells in S phase decreased concomitant with an increase in the number of cells in G2/M relative to controls. At 48 hours, knockdown of B-Myb led to increased octoploidy (8N), and the incidence of octoploidy was increased further at 72 hours (p<0.05). At this time, cells with a 16N and 32N (not shown) complement of DNA were also detectable (see arrows in the inset, Logscale). B) In these experiments, ES cells were transfected with either a NT shRNA (black line) or shRNA1 and selected by puromycin for 24 hours. Within 96 hours, the selected cells began to recover and proliferate, and within one week, cells transfected with shRNA1 demonstrated a rightward shift in the cell's DNA content, indicating that these surviving and proliferating cells were karyotypically abnormal (i.e., aneuploid). C) Metaphase chromosome spreads after labelling in the presence of BrdU for ∼24 hours and counterstaining with Giemsa. Sister chromatin exchanges (SCE) (shown by the arrows) were scored each time a color switch between dark or light sister chromatids occurred. In this study, no chromosomal abnormalities other than polyploidy or aneuploidy could be identified in any of the cells examined. D) Representative mitotic cells stained with DAPI (blue), α-tubulin (green) and γ-tubulin (red) are shown. Control (NT) mitotic cells are shown in metaphase and telophase with normal biopolar centrosomes and normal mitotic spindles. In cells lacking B-Myb, mitotic spindle and centrosome defects were readily observed. White arrows indicate cells with monopolar or multipolar centrosomes; whereas, red arrows show abnormal spindle formations. E) Graphic representation of the number of monopolar, bipolar and multipolar centrosomes for each type of nucleofected cell. *p<0.001 relative to NT controls. Bar = 10 µm.

Analysis of DNA content as a function of time revealed an even more striking phenotype. Knockdown of B-Myb decreased the number of G1 and S phase cells, increased the number of G2/M cells, and significantly increased the number of cells with >4N complement of chromosomes. Nucleofection alone, but not lipofectamine-mediated transfections, increased the incidence of cell aneuploidy in R1 cells from <0.1% to 8–11%; however, knockdown of B-Myb by either technique specifically led to either tetraploidy (4N) or aneuploidy in ∼20% of the mES cells (24 h, Figure 4A). Within 48 hours, octoploidy (8N) was observed in 9.0 to 12.5% of cells lacking B-Myb (p<0.05, n = 4), and within 72 hours, the incidence of octoploidy increased by 3-fold (NT shRNA: 11.0±1.1%; shRNA1: 30.4±2.8%; shRNA2:28.3±3.7%; n = 4). At this time, 16N cells were also readily detectable (NT shRNA: 0.12±0.04%; shRNA1: 1.69±0.44%; shRNA2: 1.76±4.1% of viable cells, n = 4)(72 h, Figure 4A), and 0.2±0.12% (n = 3) of cells had a 32N complement of chromosomes. The latter was never observed in any of the controls.

The increased incidence of polyploidy was specifically due to B-Myb knockdown and not a result of off-target effects, because when cells were co-transfected with an expression vector containing a full-length cDNA of B-Myb containing silent mutations in the sequence specifically recognized by shRNA1 (Table 1), the number of G1/S phase cells increased by 10.7±3.1% and the number of 4N cells decreased by 10.1±1.7% relative to expression vectors containing wild-type B-Myb. The number of polyploid cells generated with shRNA1 was also reduced by 27.9%; however, when the expression vector containing the silent mutation was nucleofected with shRNA2, no rescue could be observed. The rescue of the shRNA1 phenotype was however only partial, probably because knockdown of endogenous B-Myb transcripts was temporally faster than the expression of the rescue vector.

After 5–7 days of cultivation and following an initial puromycin selection for 24 hours, almost none of the surviving, proliferating cells retained 8N, 16N or 32N chromosomes. Instead, numerous cells were aneuploid. This finding was confirmed both by flow cytomety [i.e., the DNA content was shifted to the right (>2N and >4N peaks) relative to an internal control (Figure 4B)] and by chromosome spreads, where additional chromosome fragments were observed. Interestingly, the aneuploid cell population had many of the growth characteristics of karyotypically normal R1 ES cells i.e., the cells proliferated quickly, required passaging every 24–36 hours, and had similar colony morphologies. OCT4, NANOG and SOX2 proteins were also present, and when induced to differentiate, diverse cell morphologies were observed, suggesting that these aneuploid cells remained multipotent. The karyotype of some surviving cells was however abnormal (aneuploidy), leading us to conclude that these cells may no longer be viable germ-line transmissible mES cells.

To elucidate mechanisms underlying these chromosomal abnormalities, we assessed the incidence of sister chromatid exchanges (SCEs), analyzed γ-H2AX histone immunoreactivity (as an index of DNA strand breaks), and determined the number of DNA events associated with fragmentation, fusion, and breakage (abnormalities/chromosome). The incidence of SCEs and the number of abnormalities/chromosome did not differ among the groups (Table 2), and we could not demonstrate any increase in γ-H2AX histone immunostaining among any of the B-Myb knockdown groups relative to controls (n = 4). The most obvious defects in cells lacking B-Myb were numeric changes in chromosomes (Figure 4C and Table 3), which could be due to errors in spindle formation/function or chromosomal segregation during the M phase of the cell cycle. To examine these latter possibilities, R1 ES cells were immunostained with antibodies against α- and γ-tubulin, which respectively, are the major components of spindles and centrioles found in the mitotic organizing regions (centrosomes) of dividing cells. In these experiments, cells in prophase, metaphase, anaphase and telophase were readily observed in control (pSuper, NT) cells, and the majority of mitotic cells (∼80–90%) contained two centrosomes located at opposite poles in the cell. Normal spindle formation was also observed between mitotic organizing regions and chromosomes (NT, Figure 4D). In contrast, it was difficult to find any B-Myb-deficient cells in anaphase or telophase, and mitotic cells had significantly fewer bipolar centrosomes than controls (Figure 4E, p<0.001). In fact, over 35% of the mitotic cells contained monopolar spindles comprised of either a single centrosome or grouped centrosomes, and spindle abnormalities were significantly increased (B-Myb shRNA1, Figure 4D, 4E).

**Table 2:** Frequency of SCE and chromosome abnormalities

Sample I.D.	Total no. Chromosomes	SCE (SCE/Chr)	Chromosomal fragment, fusion, breakage (abnormalities/Chr)	Metaphases
pSuper	2915	872 (0.299)	10 (0.003)	69
B-Myb shRNA1	2847	1090 (0.382)	7 (0.002)	67
NT shRNA	1172	468 (0.399)	0 (0)	39

A Sister Chromatin Exchange (SCE) was scored each time a color switch between dark or light sister chromatids occurred, and chromosomal aberrations, including chromosomal breakage and fragmentation were analyzed was assessed as previously described [40]. NT- non-targeting.

**Table 3:** Frequency of polyploidy

Sample I.D.	Total metaphases	polyploidy
pSuper	89	3 (3.3%)
B-Myb shRNA	86	12 (14%)
NT shRNA	48	2 (4.1%)

The frequency of polyploidy was determined from the data gathered in the generation of Table 2.

### Loss of B-Myb promotes differentiation and apoptosis

**Figure 5:**
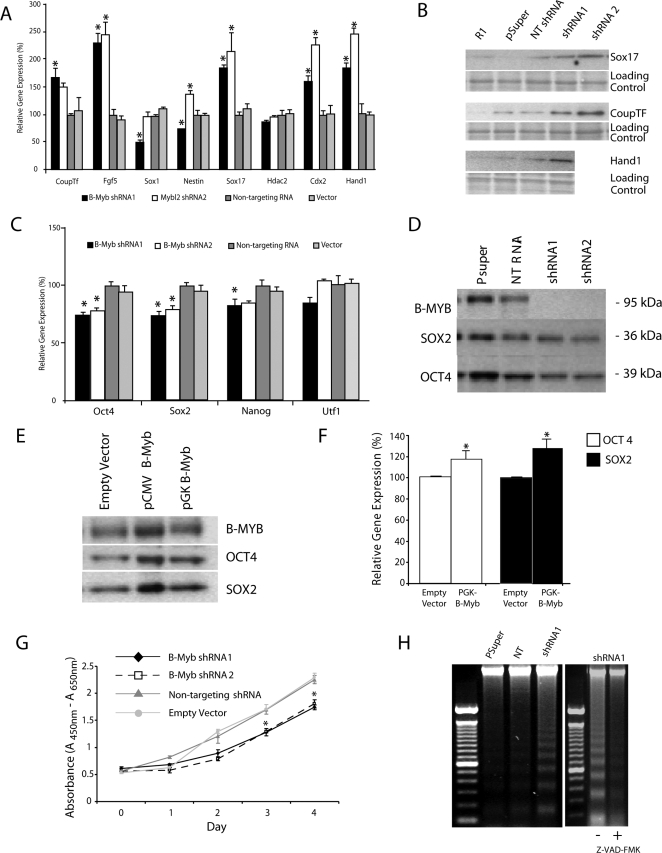
Loss of B-Myb promotes differentiation and apoptosis. A) Relative mRNA levels were determined by qRT-PCR following knockdown of B-Myb. In these experiments, markers of differentiation (CoupTf, Fgf5, Sox1, Nestin, Sox17, Hdac2, Cdx2, Hand1) were either absent or at the limits of detection in undifferentiated cells, but following knockdown of B-Myb, several markers (CoupTf, Fgf5, Sox17, Cdx2 and Hand1) showed significant (p<0.05) changes in expression. B) Western analysis of Sox17, CoupTf and Hand1 confirmed that loss of B-Myb was associated with an up-regulation in markers of differentiation. C) Knockdown of B-Myb led to a significant reduction in transcripts encoding pluripotency markers Oct4 and Sox2 within 48 hours, but not in UTF1. A significant reduction in Nanog was generally only observed >72 hours after loss of B-Myb. All data were determined by qRT-PCR. D) Knockdown of B-Myb also led to a reduction in OCT4 and SOX2 proteins at 48 hours post-nucleofection that could be detected by western (n = 3). E) Over-expression of B-MYB, relative to the parental vector control, led to increased OCT4 and SOX2 protein abundance. F) Summary data for OCT4 and SOX2 expression in mES cells following over-expression of B-Myb (PGK-B-Myb). In these experiments, the over-expression of B-Myb was transient, as were the increases in Oct4 and Sox2. G) The WST-1 assay shows a significant segregation in the absorbance of the colormetric dye in D3 cells transfected with B-Myb shRNAs relative to controls. This segregation indicates a loss in cell viability in mES cells lacking B-Myb (p<0.05). H) Loss of B-Myb promotes apoptosis, but only 72 hours after nucleofection. No increased genomic DNA fragmentation (apoptosis) was ever observed at 24 or 48 hours following knockdown. Importantly, the DNA fragmentation could be inhibited by Z-VAD-FMK, a pan-caspase inhibitor, indicating that the observed DNA laddering was due to active caspases. *p<0.05 relative to NT controls (n = 3).

Because FCS and LIF withdrawal are associated with a transient loss of B-Myb (Figures 1G and 1H) and knockdown of B-Myb led to morphological changes in mES cells consistent with differentiation (Figure 2D), we went on to assess whether knock-down of this transription factor could promote differentiation, even under cultivation conditions (+LIF and +FCS) that normally maintain pluripotency. For this, we quantified RNAs encoding early markers of differentiation (CoupTF and FGF5), and markers specific for ectoderm (Sox1, Nestin), endoderm (Sox17, Gata4), mesoderm (Brachyury), and trophectoderm (Cdx2, Hand1) lineages (Figure 5A). Under control conditions, these markers were either undetectable or at the limits of PCR detection, but within 48 hours of knocking down B-Myb, CoupTF and FGF5 were significantly up-regulated, as were both trophectoderm markers (p<0.05, n = 4). Definitive endoderm markers Sox17 and Gata4 (not shown) mRNAs were upregulated; however, brachyury was undetectable in cells at all time points examined, and the neuro-ectoderm markers gave variable results. A non-significant decrease in Hdac2 was also observed. The up-regulation of some differentiation markers (COUPTF, SOX17, and HAND1) was furthermore confirmed by western (Figure 5B), but their presence was only observed at >48 hours post-nucleofection.

Importantly, a significant decrease in *pou5f1* and *sox2* gene products (RNA and protein)(Figures 5C, 5D) was observed within 24–48 hours of B-Myb knock-down, while transient over-expression of B-MYB increased endogenous OCT4 and SOX2 proteins by 18–30% (p<0.05, Figure 5E, 5F). In contrast, no consistent decrease in NANOG could be demonstrated after knock-down of B-Myb except in puromycin- selected cells between 48 and 72 hours after transfection. Over-expression of B-Myb did not produce a significant increase in NANOG. These data in particular suggest that the genes encoding Sox2 and Oct4, but not Nanog, might be targets of B-MYB regulation.

To further validate the findings of differentiation, independent of gene markers, we examined the viability of the transfected cells and determined whether apoptosis, which is generally elevated with differentiation, was altered. For this we employed the the water-soluble tetrazolium (WST)-1 assay, an indicator of both mitochondrial function in living cells and cell viability. Using this assay, we found a significant segregation and loss of viability in mES cells transfected with B-Myb shRNAs (p<0.05, Figure 5G). Because no increase in apoptosis, measured either by annexin V (a Ca^2+^-dependent, phospholipid binding protein with a high affinity for phosphatidylserine) and propidium iodine (a nuclear stain) staining (data analyzed by flow cytometry, not shown) or by DNA fragmentation (i.e., DNA laddering), could be demonstrated at 24 or 48 hours post-nucleofection, the loss of viability appeared to be wholly due to reduced cell proliferation; however, at 72 hours post-transfection, DNA fragmentation with a pattern typical of programmed cell death was readily detectable (n = 3 independent preparations). The DNA laddering was caspase dependent, and it could be fully inhibited by addition of 40 µmol/L Z-VAD-FMK (carbobenzoxy-valyl-alanyl-aspartyl-[O-methyl]-fluoromethylketone) a cell-permeant pan caspase inhibitor (Figure 5H). Moreover, activated caspase 3 was observed at >48 hours in those cells nucleofected with shRNA1 (data not shown); however, activated caspase 3 could not be detected in controls (pSuper, NT) at any time point examined. Importantly, the increase in DNA laddering and in caspase 3 was only significantly increased in cells following knock down of B-Myb (p<0.05).

### B-Myb modulates the *pou5f1* gene promoter

**Figure 6:**
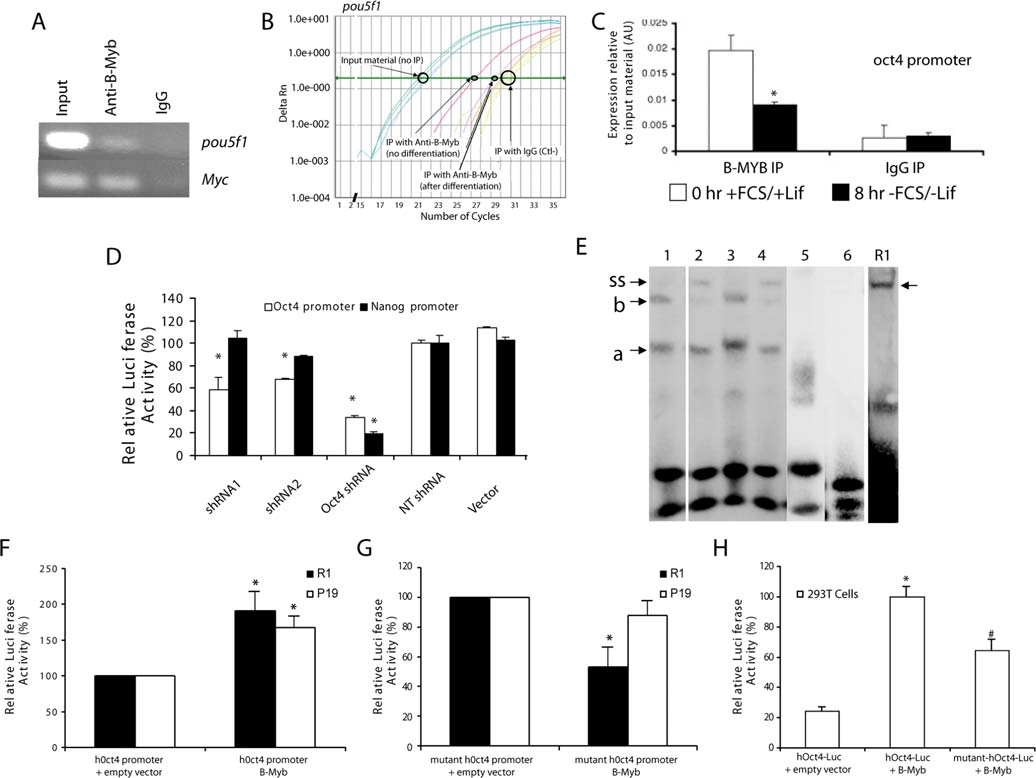
B-Myb binds to and regulates Oct4 promoter activity. A) Chromosomal immunoprecipitation (ChIP) assays were performed with extracts from R1 mES cells. In these experiments, PCR amplifications were performed with total input DNA (positive control without immunoprecipitation) and following immunoprecipitation (IP) with antibodies to either B-MYB or to rabbit IgG (as a negative control). Amplifications of immunoprecipitated DNA were analyzed by agarose gel electrophoresis, and Myc was employed as a positive control. The presence of an amplification product in the anti-B-Myb immunoprecipitated DNA indicates that B-Myb binds a proximal region of the mouse *pou5f1* promoter. B) In the second set of experiments, which were evaluated by qRT-PCR, we find that B-MYB dynamically binds to the endogenous *pou5f1* promoter region. In undifferentiated cells, an amplification signal 10–15-fold above that seen in the IgG control is observed; however, this signal is reduced to only 2–4 fold above the IgG control following withdrawal of LIF and FCS. All qRT-PCR amplifications were performed using SYBR Green with primers located 5′ and 3′ to the putative binding site. C) The dynamic decrease in chromatin binding of B-Myb to the *pou5f1* gene promoter in mES cells cultivated either in the presence or absence of FCS and LIF is illustrated graphically (p<0.05, n = 3). D) To test the transactivation potential of B-MYB on pluripotency genes, human (h)Oct4 and mouse Nanog promoter constructs containing a Luciferase reporter were used in transient transfection assays. In these experiments, D3 cells were transfected with the constructs as indicated. The positive control Oct4 shRNA reduced the promoter activities of both the hOct4 and mouse Nanog promoters; however, knockdown of B-Myb only reduced the promoter activity of the hOct4 promoter. *p<0.05 relative to NT controls. E) To determine if B-MYB proteins could bind to the human Oct4 promoter, EMSAs were performed with HeLa cell nucleoextracts (lanes 1–6) and R1 nucleoextracts. Positive control cassettes revealed two DNA-protein complexes (*a* and *b*) in HeLA cell nuclear extracts and a single higher molecular weight complex in extracts from R1 ES cells. Addition of anti-B-MYB antibodies resulted in formation of a complex in mouse with a lower mobility, and in HeLa cells, most but not all of complex *b* (supershift, ss) showed decreased mobility. Complex *a* did not show any change in mobility and was therefore considered non-specific. An oligonucleotide cassette containing base pair changes within the putative MYB-binding site failed to form DNA complexes in either R1 or HeLa cells, and the addition of a 200-fold molar excess of unlabeled competitor oligonucleotide also led to a loss of signal. [Data are presented as follows: Lane 1: biotin-end labeled 24 bp DNA duplexes containing the putative MYB-binding site located at position –223 of the hOct4 promoter. Lane 2: same as in Lane 1, but with the addition of anti-B-MYB antibodies. Lane 3: biotin-end labeled 27 bp DNA B-Myb consensus sequences (positive controls). Lane 4: as Lane 3 plus anti-B-MYB antibodies. Lane 5: as Lane 1, with non-labeled competitor DNA (200-fold). Lane 6: as Lane 1, with biotin-labeled DNA duplexes containing a mutated Myb binding sequence. R1: biotin-end labeled 24 bp DNA duplexes containing the putative MYB-binding site located at position –223 of the hOct4 promoter and incubated with nucleoextracts from R1 ES cells. Note that the DNA protein complex migrates more slowly than those observed with HeLa cells.] Oligonucleotide sequences are described under Methods. F) A 2.8 kb hOct4 promoter-Luciferase construct was employed to assess the ability of B-MYB to directly regulate its activity in vitro. In these experiments, R1 ES and P19 EC cells were co-transfected with plasmids containing a 2.8 kb hOct4 promoter-Luciferase construct promoter and either a CMV promoter driven mouse B-Myb construct or empty vector. Relative luciferase activity, normalized to Renilla luciferase and the empty vector controls, was taken as a measure of transcriptional activity. G) R1 ES and P19 EC cells were co-transfected with either a CMV promoter driven mouse B-Myb construct or empty vector, but in this case, the 2.8 kb hOct4 promoter-Luciferase construct contained a mutation in the putative Myb-binding site (position –223) that had been putatively identified from a genomic analysis with Genomatix software. H) To exclude the possibility that pluripotency factors present in ES and EC cells might have affected the relative luciferase activities, wildtype and mutant hOct4-Luc constructs were co-transfected into human kidney 293T cells. In these experiments, the relative activity of the hOct4-Luc constructs was much less than that observed in R1 or P19 cells; however, when co-tranfected with the CMV-B-Myb vector, the relative luciferase activity increased significantly relative to empty vector controls. The increase was however significantly less when the mutant hOct4-Luc construct was employed in 293T cells. In summary, data from the luciferase assays (F, G and H) indicate that B-Myb can transactivate the hOCT promoter in vitro, and that its actions are mediated in part through protein binding to the proximal Myb-binding site located at position –223. * p<0.05 relative to either the native or mutant hOct4 promoter-Luciferase+empty vector. #p<0.05 relative to mutant hOct4-Luciferase+B-Myb (PGK-BMYB).

Having determined that alterations in B-Myb led to corresponding changes in OCT4 protein abundance, the promoter region of the *pou5f1* gene was examined to identify possible MYB binding sites. In mouse, six putative *mybl2* binding sites (consensus and non-consensus) were identified with software from Genomatix (http://www.genomatix.de/). Consensus binding sites corresponded to sequences at positions –295 (CAACaG), -799 (-CAACgG), -1454 (CAACaG), and –2251 (TAACtG). To determine if any of these sites might bind B-MYB, chromosomal immunoprecipitation (ChIP) assays were performed with DNA extracts from mES (R1) cells. Since B-MYB had previously been shown to bind to the *myc* promoter, it was employed as a positive control [16], [17]; while, IgG antibodies were employed as a negative control. Following immunoprecipitation with an antibody to B-MYB, an amplification product was observed both from the *myc* promoter and from the *pou5f1* gene promoter (Figure 6A); however, no amplification product could be observed following immunoprecipitation with IgG. Moreover, differentiation of ES cells by withdrawal of FCS and LIF led to a significant decrease in the SYBR Green signal observed by qRT-PCR from the *pou5f1* gene promoter following B-MYB immunoprecipitation (Figure 6B, 6C). Since these results are consistent with the differentiation-associated transient loss of B-MYB in ES cells shown in Figures 1H and 1I, these data confirm the specificity of these assays and show that B-Myb dynamically binds to the *pou5f1* gene promoter in ES cells.

Next, we employed the mouse nanog and human Oct4 promoters in transient transfection assays of murine ES cell lines (D3, R1) to determine whether B-MYB could directly regulate the transcriptional activity of pluripotency genes. In these experiments, knockdown of B-Myb significantly decreased Oct4 promoter activity by 30–40% relative to controls (NT and pSuper); whereas, Nanog promoter activity did not significantly differ among any of the experimental groups (i.e, NT, pSuper, shRNA1 and shRNA2). In contrast, shRNA knockdown of endogenous Oct4 transcripts significantly reduced the activities of both the hOct4 and Nanog promoters by ∼70 and 80%, respectively (Figure 6D), confirming that effects of B-Myb were specific to the hOct4 promoter.

Similar to mouse, the human Oct4 promoter sequence contained 6 putative Myb binding sites [position: -233 (CAACtG), -787 (CAACaG), -1046 (TAACaG), -1239 (non-consensus: AACGgG) -1898 (CAACgG) and –2378 (TAACtG)]; however, only one of these, at position –233, was located within the proximal promoter near a consensus sequence present in the mouse gene. An electrophoretic mobility shift assay (EMSA) was therefore employed to determine if B-MYB could bind to this site. For comparative purposes, a consensus MYB-binding sequence from the *bcl2* gene was used as a positive control [18]. In these experiments, double stranded oligonucleotide cassettes were incubated with nuclear extracts from HeLa cells or mouse R1 cells. The positive controls showed protein binding, and two DNA-protein complexes (*a* and *b*) were identified with HeLa cell nuclear extracts (Figure 6E), however, a single higher molecular weight complex was observed with extracts from R1 ES cells, suggesting that the transcriptional complexes differed between these two cell lines. The addition of anti-B-MYB antibodies resulted in formation of a complex in mouse with a lower mobility, and in HeLa cells, most but not all of complex b (supershift, ss) showed decreased mobility. Complex *a* did not show any change in mobility and was therefore considered non-specific. The specificity of these protein-DNA complexes for B-Myb was further verified by an oligonucleotide cassette containing base pair changes within the putative MYB-binding site, which failed to form DNA complexes in either R1 or HeLa cells, and by addition of a 200-fold molar excess of unlabeled competitor oligonucleotide.

To determine whether the myb-binding site was functional, pluripotent murine R1 ES, P19 EC and non-pluripotent human embryonic kidney 293T cells were transfected with native and mutant hOct4-Luc constructs together with either a B-Myb over-expressing vector under the control of the phosphoglycerate kinase promoter or an empty vector. All luciferase data are reported relative to the empty vector and after normalization to Renilla luciferase activity. In both R1 ES and P19 EC cells, transient over-expression of B-MYB led to an increase in hOct4-Luciferase activity (Figure 6F). No significant increase in luciferase activity could be demonstrated in P19 cells relative to controls, but an ∼50% decrease in luciferase activity was observed in R1 ES cells (Figure 6G), with the mutant constructs relative to controls. These data suggested differential gene regulation between ES and EC cells, consistent with what is known about the Oct4 promoter [19].

To rule out any possible effects associated with endogenous pluripotency transcription factors (i.e., Oct4 or Sox2) present in ES and EC cells, 293T cells were transfected with hOct4-Luc constructs. In these experiments, normalized promoter activities of mutant and wild-type constructs were much less in 293T cells than in ES cells (p<0.05), and no difference was observed between 293T cells co-tranfected with either the mutant or wildtype hOct4-promoter and the empty parental vector. Over-expression of B-Myb, however, selectively enhanced wildtype hOct4-luciferase activity by >3–5-fold (Figure 6H). Unexpectedly, the mutant hOct-4-Luc constructs also showed an increase of ∼1.5–2-fold, suggesting that another binding site on this promoter might be regulated by B-MYB. The increase observed in the mutant was however significantly less than that elicited by the wildtype hOct4 promoter sequence, leading us to conclude that the *trans*-activation potential of the human Oct4 promoter by B-MYB is elicited, at least in part, through the MYB-binding site located in the proximal promoter of this gene.

## Discussion

**Figure 7:**
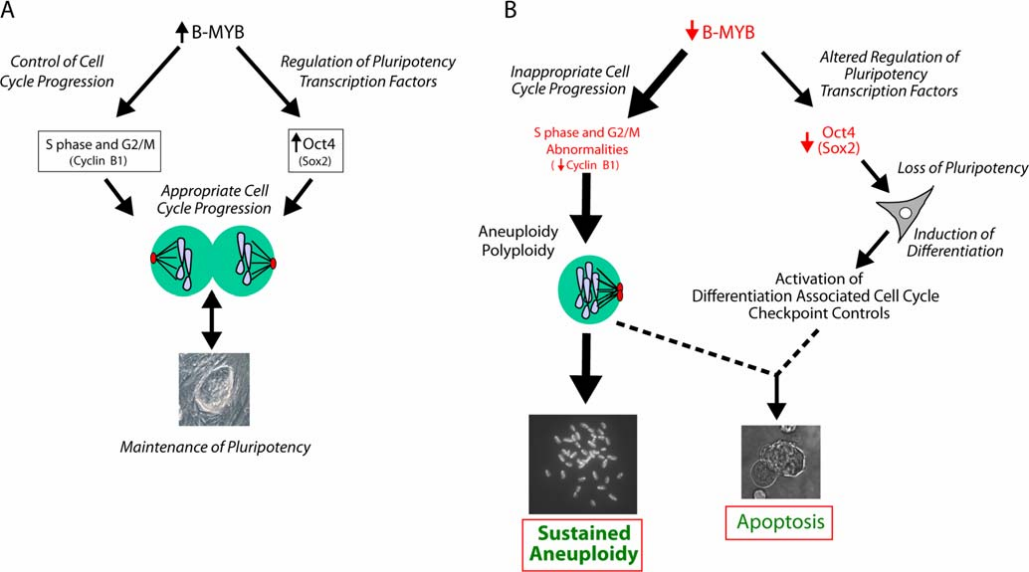
Proposed functions of B-Myb in ES cells. A) Under normal conditions, high levels of endogenous B-Myb promote proliferation through coordination of events in S phase and appropriate transit through G2/M. It also modulates Oct4 expression to help maintain the undifferentiated and pluripotent state of ES cells. B) The primary effect of reduced levels of B-Myb is the generation of polyploid and aneuploid cells due to a loss of normal cell cycle progression through G2/M and spindle defects. Loss of B-Myb also causes a reduction in Oct4 expression and ES cell differentiation. Because differentiated cells have tight checkpoint controls that are capable of recognizing chromosomal abnormalities and activating apoptosis, this may represent a control mechanism necessary to maintain chromosome fidelity during early embryo development. Many of the surviving mES cells that were transiently deficient in B-Myb do not undergo apoptosis. Instead some of these cells become aneuploid, and can no longer be considered as authentic ES cells. Gene products in parentheses represent additional but putative targets of B-Myb regulation.

In the present study, we show that knockdown of B-MYB in mES cells decreases proliferation, cell viability and colony size, and it delays transit through the G2/M phase of the cell cycle. The latter is associated with centrosome and mitotic spindle defects, which lead to a high incidence of polyploidy that results either in programmed cell death or sustained aneuploidy (see Figure 7). The activation of apoptosis, however, only occurs after the induction of differentiation and subsequent to the loss of pluripotency factors.

Iwai et al. previously found that a dominant-negative interfering form of C-Myb (dnMyb) led to an accumulation of mES cells in the G1 phase of the cell cycle as well as to changes in proteins associated with cell adhesion [15]. They showed that activation of dnMyb did not induce the expression of any germ layer differentiation markers, even though E-Cadherin, a marker of epiblast, was down-regulated; and they did not observe any apoptosis. One possible explanation for the disparity between their study and ours may be experimental. In the present study, we employed shRNAs and puromycin selection to examine cells that had a significant and specific reduction in B-MYB proteins. In contrast, Iwai et al. had assumed, because of the conserved protein homologies and consensus binding sequences, that the dominant-negative interfering form of C-Myb would inhibit the activity of B-Myb in ES cells; however, important differences are now known to exist between these two transcription factors. First, only B-Myb, and neither A- nor C-Myb, can rescue the phenotype of Dm-Myb deficient Drosophila mutants in complementation experiments [6]. Second, transactivation of genes by *myb* family members varies considerably depending on the cell type and promoter context [1], and in the absence of co-factors, for example, B-MYB is a relatively poor trans-activator of some gene promoters when compared with either C-MYB or A-MYB. Third, phylogenetic analyses imply that the A*-myb* and C-*myb* genes arose from a B-*Myb*/Dm*-Myb*-like ancestor by two gene duplication events that included the acquisition of a unique transcriptional activation domain [6]. It is therefore likely that knockdown of B-Myb would have different effects on either transcription and/or replication than those exhibited by a dominant-negative interfering form of C-Myb.

Our results also provide an additional explanation for the causes of embryonic lethality reported by Tanaka et al. [14]. Developmentally and after implantation on day 4, the early murine epiblast begins to expand and differentiate to form columnar epithelium. This transition is accompanied by increased cell proliferation and expansion of pluripotent cells from ∼25 to ∼660 cells by 6.5 dpc [20]. Although the embryonic lethality of B-Myb-knockout mice at 4.5–6.5 dpc has generally been attributed to defects in proliferation, we show that the primary cell cycle defects in mES cells are due to delays in G2/M that are associated with mitotic spindle defects and polyploidy. We also show that loss of B-Myb in LIF-cultivated ES cells leads to an up-regulation of differentiation markers followed by an activation of caspase-mediated DNA fragmentation. Since mES cell differentiation is associated with the establishment of robust cell cycle checkpoint controls that strongly promote apoptosis of polyploid cells [21], the loss of ICM observed by Tanaka et al. may have resulted not only from a proliferation defect but also from programmed cell death that is activated in response to differentiation and the formation of columnar epithelium.

Moreover, the data presented in the current paper are consistent with B-Myb being a general regulator of S phase progression. In fact, we show highly significant and inappropriate BrdU incorporation into chromatin (Figure 3C), which indicates either a potential replication defect or problems with chromosome condensation. Similarly, Manak et al. have shown in Drosopholia that the primary function of B-MYB is associated with DNA replication, and not in gene regulation [11], [12]; however, we also show specific defects in mitotic progression and spindle formation that are similar to those reported in zebrafish. Shepard et al. showed that zebrafish mutants lacking *crb* (the zebrafish B-myb equivalent) had enhanced chromosomal instability and that injection of cyclin B RNA partially restored the genomic integrity of Myb mutants [13]. Consistent with their findings, cyclin B1 transcripts are decreased in ES cells following knockdown of B-Myb (unpublished data), and since cyclin B is activated in late S phase by B-Myb, it is likely that the defects in spindle formation and centrosomes observed in ES cells lacking B-Myb are elicitied, at least in part, through control of cyclin gene expression during progression through S phase.

Importantly, B-MYB specifically binds to a proximal sequence in the endogenous mouse *pou5f1* promoter, and altered expression of B-Myb in mES cells leads to coordinate changes in endogenous Oct4 expression. Although the changes in endogenous Oct4 expression are modest, studies with the human Oct4 gene promoter show that the effects of B-Myb on this gene are unambiguous. Specifically, point mutations to a proximal myb-binding site of the human Oct4 promoter inhibited the trans-activating potential of this transcription factor both in pluripotent stem and 293T cells; however, the inhibition in 293T cells, was only partial, suggesting that another binding site or formation of an unique transcriptional complex regulated by B-MYB may be involved in the control of *pou5f1* gene activity. Some differences in activity were also noted between R1 ES and P19 EC cells (see Figure 6D), and since the Oct4 promoter depends on distal and proximal enhancer elements to control pre- (ES cell) and post- (EC cell) implantation expression, respectively, a detailed promoter analysis will be required to elucidate how *pou5f1* promoter activity is regulated by B-MYB [19]. It is likely, however, that the effects of B-Myb on the *pou5f1* gene promoter will be primarily modulatory. The myb-binding site that was mutated in this study is not present within any of the four conserved regions of homology or enhancer regions known to be critical to its regulation [19]. Over-expression of B-Myb also appears insufficient to prevent differentiation following LIF withdrawal [22]. It is however possible that some fate decisions (i.e., Cdx2, Hand1 expression) may be mediated, in part, through B-Myb mediated changes in Oct4 expression, particularly since a modest change in OCT4 can promote ES cell differentiation to trophectoderm [23] or specification to a cardiac lineage [24]. Because several pluripotency-associated genes [Fgf4, c-Myc, Bcl-2] are also regulated by B-Myb in non-ES cells [16]–[18], [25]–[28], their potential effects secondary to changes in B-Myb function on fate decisions, specification and differentiation of ES cells can not be ignored. Future experiments will be required to deal with these possibilities.

In conclusion, B-Myb is required for maintenance of the pluripotent state, and its expression and function are critical to and potentially a determining factor of murine ES cell fate decisions in vitro. Appropriate levels of B-Myb are required to maintain chromosomal integrity and cellular euploidy, and any dysregulation in the expression or function of B-Myb promotes differentiation and/or aneuploidy that can result in apoptosis (Figure 7). B-Myb is therefore critical not only for mouse embryonic development, but also for a normal karyotype and maintenance of viable embryonic, and potentially, all proliferating somatic stem cells. Because human ES cells are susceptible to chromosomal rearrangements and karyotypic abnormalities, the data generated in this study suggest one possible mechanism that may account for some of this instability.

## Materials and Methods

### Cell Culture

#### Mouse and Human cell lines

D3, R1, E14 murine (m)ES cell lines, H1 (h)ES (WiCell, Agreement No: 05-W102, NIH code WA01) cells, P19 EC and EG-1 cell lines were cultivated on feeder layers of mouse embryonic fibroblasts (MEFs) or under feeder free conditions as previously described [29]–[31]. HeLa and 293T cells (ATCC) were grown in DMEM supplemented with 10% fetal bovine serum (Invitrogen), and Sca1-positive cells, lineage-negative cells, were isolated from bone marrow (BM) of CD-1 mice after passage over a Spin-Sep Murine cell column (Stem Cell Technologies, Cat No 17036).

### RNA Preparation, Reverse Transcription, and Real-Time PCR Analysis

RNA was extracted, treated with DNAse, and cDNA synthesized using High Capacity cDNA Archive Kit (Applied Biosystems). Real-time PCR reactions were performed with an ABI PRISM 7900HT Sequence Detector System (PE Applied Biosystems) using a SYBR Green protocol or TaqMan protocols with the core reagent kit and either SYBR Green PCR Master Mix (Applied Biosystems) or Platinum® SYBR® Green qPCR SuperMix (Invitrogen**)** [32]. RNA from TS cells was kindly provided by M. Ko (NIA, Baltimore). Primers were designed with Primer Express 2.0 software (Table 4) or pre-designed by Applied Biosystems (TaqMan® Gene Expression Assays).

**Table 4:** Primers used for qRT-PCR analyses

Target	Primer
Nanog	FW: TTTCAGAAATCCCTTCCCTCG
	RV: CGTTCCCAGAATTCGATGCT
A-Myb	FW: CGCGCCTATGCGGTACTT
	RV: TCAGCATATTGAAGGTCATCATCCT
B-Myb	FW: GTGAGGCAGTTTGGACAGCAA
	RV: GGATTCAAAACCCTCAGCCA
C-Myb	FW: GTGAGGCAGTTTGGACAGCAA
	RV: GGATTCAAAACCCTCAGCCA
Oct4	FW: CAATGCCGTGAAGTTGGAGA
	RV: GCTTCAGCAGCTTGGCAAAC
H1F0	FW: AGATCGCGAGTCAGGTTCTG
	RV: GTGGAGTTCTCGGTCATGGT

Histone 1 (H1F0) was used as an internal control for PCR reactions for normalization purposes. Pre-designed primers were also purchased from Applied Biosystems (TaqMan® Gene Expression Assays) for detection of the following transcripts: Sox1, Utf1, CoupTf, Fgf5, Nestin, Sox17, Hdac2, Actin, Cdx2, Hand1.

### Protein Analysis

Westerns were performed as described [32], [33], and membranes probed with primary antibodies to rabbit B-myb (Abcam and Santa Cruz), rabbit Nanog, rabbit cyclin B1 (Abcam), rabbit A-myb, rabbit C-Myb, goat Cdx2, rabbit CoupTF, rabbit eHand, goat Fgf5, goat Sox1 and goat Sox17 (Santa Cruz), rabbit active Caspase-3 (Cell Signal), and rabbit Oct4 (Chemicon). Blots were re-probed with HRP conjugated goat anti-rabbit IgG(H+L) (Zymed) or donkey anti-goat IgG(H+L) (Santa Cruz), and horseradish peroxidase was detected using Pierce Super Signal ECL substrate kit and chemiluminescence captured on Kodak BioMax Light film. Immunostaining of mouse ES cells was performed as described [29], [32].

### Plasmids containing shRNAs and B-Myb

Nineteen-base-pair gene-specific regions for RNA interference were designed based on algorithm rules by [34], [35]. Oligonucleotides were cloned into pSUPER.puro (Oligoengine) after BLAST analysis to ensure against sequence similarity with other genes. [36].

B-Myb was amplified using primers GAGCAGCCTGAGTCCTGACC and AGACCCTGATAGGGTTCCTTCT found at positions −87 and +2367, respectively of Mybl2 sequence *NM008652*. The cDNA was cloned into the parental (CMV promoter) or a modified pIRES2-EGFP expression vector (BD Bioscience Clonetech) containing a 554 bp PGK1 promoter. The mouse B-Myb cDNA was mutated by PCR with primers GGTGTGACTTAAGTAAA (fw) and TTTACTTAAGTCACACC (rev).

### Transfections/Nucleofections

ES cells were transfected with lipofectamine using 1.5 µg of shRNA plasmids in 12-well plates. Selection with 1 µg/ml of puromycin (Sigma) was performed 24h post-transfection for a period of three days to enrich for transfectants. Plasmid DNA was also introduced into ES cells using the Nucleofector mouse ES Cell Kit from Amaxa Biosystems (Cologne, Germany Cat. No VPH-1001) according to the manufacturer's instructions and selected with puromycin where indicated.

### Cell Cycle Analysis

DNA cell cycle analysis was measured on propidium iodide (10 µg PI, Sigma)-stained nuclei using a FACSCalibur. Cell cycle compartments were deconvoluted from single-parameter DNA histograms of 10,000 cells. 5-Bromodeoxyuridine (BrdU) incorporation was measured in cells pulsed (30 min) with 10 µM BrdU (Sigma) as described [37], using a FITC-labeled monoclonal antibody (0.2 µg/mL) to BrdU (clone B44, Becton Dickinson).

### Luciferase Reporter Constructs and Assays

A 3 kb human *pou5f1* promoter and a 300 bp mouse *nanog* promoter cloned upstream of the firefly luciferase gene were employed [38]. Cells were seeded at a density of 30,000/well in 96-well plates, and reporter (75 µg), shRNA (500 ng), and pRL-SV40 *Renilla* luciferase plasmid (5 ng)(Promega), as a transfection control, were transfected into cells with Lipofectamine 2000 (Invitrogen). Firefly and *Renilla* luciferase activities were measured 24 or 48 h post-transfection with Dual Luciferase System (Promega) on a Centro LB960 (Berthold Technologies) or an Applied Byosystem TR 717 Microplate Luminometer. For over-expression studies with PGK (or CMV)-B-Myb, data were normalized relative to renilla luciferase (internal transfection control) and to pGL3 luciferase to facilitate cell line comparisons.

### Sister Chromatid Exchange

To estimate sister chromatid exchanges (SCE) as a parameter of recombinatorial events, sister chromatids of the metaphase chromosomes were differentially stained after mitotic division as described [39]. A SCE was scored each time a color switch between dark or light sister chromatids occurred, and chromosomal aberrations, including chromosomal breakage and fragmentation was assessed as previously described [40].

### Chromatin Immunoprecipitation (ChIP)

Chromatin immunoprecipitation (ChIP) was performed as described in the EZ-Chip Kit (Catalog #17–371, Upstate). Briefly, mouse ES cells were cross-linked in 1% formaldehyde in phosphate-buffered saline (PBS), and lysed in the presence of a protease inhibitor cocktail. The chromatin was sheared to an average of 200–800 bp, following sonication on ice using the Sonic Dismembrator 100 (Fisher). Immunoprecipitation and DNA recovery were performed overnight. Antibodies to rabbit IgG (Santa Cruz, Cat No. sc-2027) were employed as a negative control for immunoprecipitation. Input DNA and immunoprecipitated DNA were analyzed by PCR [GC-Rich PCR system (Roche)] for binding of B-MYB.

### EMSAs

Nucleoextracts from R1 ES cells were prepared with the CelLytic™ NuCLEAR™ Extraction Kit (Sigma) or purchased (Hela Nuclear Extract, Upstate Biotechnology, USA). EMSAs were performed with **a** LightShift Chemiluminescent EMSA Kit (Pierce, USA) according to the manufacturer's instructions. Oligonucleotide sequences were as follows: hOct4 Putative B-Myb binding site - CGGGAGACA**CAAC**TGGCGCCCCTC; Mutant binding site: CGGGAGACA**ATTA**TGGCGCCCCTC; and consensus B-Myb binding site - AGATGTCGCCCCTGGTGGA**CAACAT**CG [18]. Supershift assays were performed by adding 1 µg of a polyclonal antibody to B-Myb (Santa Cruz Biotechnology, Inc), and competitions were performed in the presence of a 200-fold excess of unlabeled oligonucleotide DNA cassette.

### Cell Viability Assay and Apoptosis

ES cells, seeded at a density of 10,000 and 15,000/well in a 96-well plate, were transfected with 0.5 µg of shRNA plasmid. The WST-1 cell proliferation assay kit (Roche) was employed according to the manufacturer's instructions with 10 µl of WST reagent in 100 µl of medium per well. For apoptosis, fragmented genomic DNA was detected following size fractionation on agarose gels, and all measurements were normalized to total cellular protein [41]. The proportion of apoptotic cells was also determined using Annexin V-FITC Apoptosis Kit (BD Biosciences) by fluorescence-activated cell sorting (FACS) performed on a FACSVantage (BD Biosciences).

### Statistical Analyses

Results are presented as mean±S.E.M. Non-parametric statistical analyses, including the Mann-Whitney U test or the Kolmogorov-Smirnov two-sample test, and analyses of variance (ANOVA) followed by multiple comparisons of means by a Student-Newman-Keuls' procedure were used to evaluate differences between means of experimental groups. A Chi-square test was used to determine differences with categorical values. P values less than 0.05 were considered as statistically significant.
